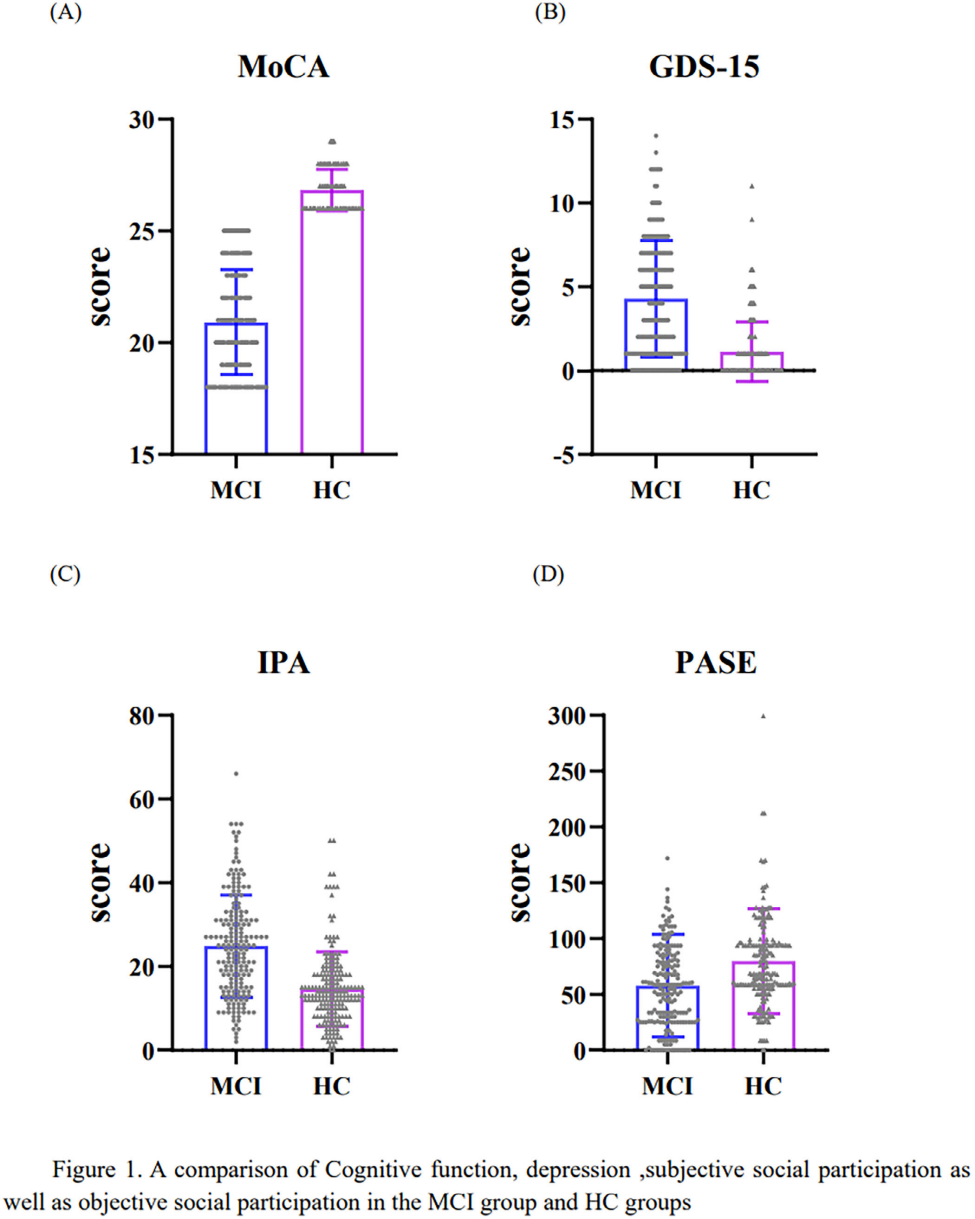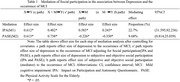# The Mediating Role of Social Participation in Depression and the Occurrence of Mild Cognitive Impairment among the Elderly in China: a prospective, case control, cross‐sectional study

**DOI:** 10.1002/alz.084458

**Published:** 2025-01-09

**Authors:** Xiaoyuan Qiu, Lifeng Zhang, Yifan Ye

**Affiliations:** ^1^ School of Nursing, Sun Yat‐sen University, Guangzhou, Guangdong China; ^2^ Sun Yat‐sen University Cancer Center, Guangzhou, Guangdong China

## Abstract

**Background:**

Depression is common in the elderly, which is regarded as is an important risk factor for mild cognitive impairment (MCI), and social participation has been proved to be effective in improving the mental health of the aged, while the relationship and acting path among depression, social participation and the occurrence of MCI are still unclear.

**Method:**

This study included 190 older adults with MCI and 190 matched older adults without cognitive impairment. All participants were asked to complete Montreal Cognitive Assessment (MoCA), the Participation and Autonomy Questionnaire (IPA), the Physical Activity Scale for the Elderly (PASE) and the Short Form Geriatric Depression Scale (GDS‐15). Binary regression and linear regression were used to investigate the relationship among depression, MCI and social participation. And RMediation was used to test mediating effect of social participation between depression and MCI.

**Result:**

The occurrence of MCI was associated with more severe depression symptom (*OR* = 1.542,*95%CI* = 1.350 to 1.743), lower subjective and objective social participation level (*OR* = 1.204,0.987; *95%CI* = 1.099 to1.319, 0.897 to 0.890), and those with more severe depression symptom showed lower subjective and objective social participation level (*β* = 0.582,‐0.229;*95%CI* = 1.832 to 2.467,‐4.981to ‐1.801)). RMediation analysis showed that both subjective and objective social participation played a significant role in mediating the depression and the occurrence of MCI (*95% CI* of a*b = 31.595 to 92.236, *95% CI* of a*b = 0.814 to 25.039), and the indirect effects were 22.7% and 9.68%, respectively.

**Conclusion:**

These findings imply that depression impacts MCI directly and indirectly through social Participation. Subsequent interventions should focus on improving social participation in the elderly to achieve greater outcomes for MCI management.